# 2-(4-Bromo­phen­yl)acetohydrazide

**DOI:** 10.1107/S1600536812027006

**Published:** 2012-06-30

**Authors:** Shakeel Ahmad, Abdul Jabbar, Muhammad Tahir Hussain, M. Nawaz Tahir

**Affiliations:** a Department of Chemistry, Government College University, Faisalabad 38000, Pakistan; b Department of Applied Sciences, National Textile University, Faisalabad 37610, Pakistan; c University of Sargodha, Department of Physics, Sargodha, Pakistan

## Abstract

In the title compound, C_8_H_9_BrN_2_O, the 1-bromo-4-methyl­benzene group and the formic hydrazide moiety [r.m.s. deviations of 0.0129 and 0.0038 Å] are oriented at a dihedral angle of 80.66 (11)°. In the crystal, mol­ecules are linked *via* strong N—H⋯O hydrogen bonds, leading to the formation of chains in the [010] direction. These chains are linked *via* weaker N—H⋯N and N—H⋯O hydrogen bonds, with *R*_2_^2^(7) and *R*_3_^2^(7) ring motifs, forming a two-dimensional network parallel to (001).

## Related literature

For background literature and the crystal structure of 2-chloro­benzohydrazide, see: Ahmad *et al.* (2012 ▶). For graph-set notation, see: Bernstein *et al.* (1995 ▶).
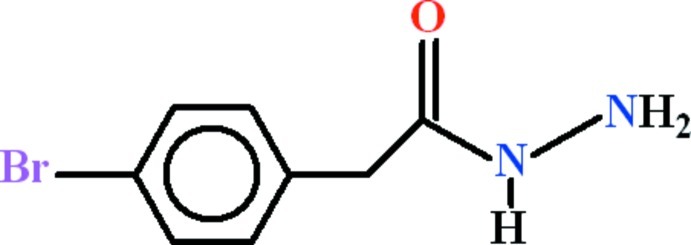


## Experimental

### 

#### Crystal data

C_8_H_9_BrN_2_O
*M* *_r_* = 229.07
Monoclinic, 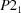

*a* = 6.0798 (2) Å
*b* = 4.8565 (1) Å
*c* = 15.1126 (5) Å
β = 98.003 (2)°
*V* = 441.88 (2) Å^3^
*Z* = 2
Mo *K*α radiation
μ = 4.60 mm^−1^
*T* = 296 K
0.36 × 0.23 × 0.22 mm

#### Data collection

Bruker Kappa APEXII CCD diffractometer
Absorption correction: multi-scan (*SADABS*; Bruker, 2009 ▶) *T*_min_ = 0.298, *T*_max_ = 0.366
4331 measured reflections
1814 independent reflections
1677 reflections with *I* > 2σ(*I*)
*R*_int_ = 0.023

#### Refinement

*R*[*F* ^2^ > 2σ(*F* ^2^)] = 0.023
*wR*(*F* ^2^) = 0.059
*S* = 1.06
1814 reflections
115 parameters
1 restraint
H atoms treated by a mixture of independent and constrained refinement
Δρ_max_ = 0.44 e Å^−3^
Δρ_min_ = −0.47 e Å^−3^
Absolute structure: Flack (1983 ▶), 583 Friedel pairs
Flack parameter: 0.007 (11)

### 

Data collection: *APEX2* (Bruker, 2009 ▶); cell refinement: *SAINT* (Bruker, 2009 ▶); data reduction: *SAINT*; program(s) used to solve structure: *SHELXS97* (Sheldrick, 2008 ▶); program(s) used to refine structure: *SHELXL97* (Sheldrick, 2008 ▶); molecular graphics: *ORTEP-3 for Windows* (Farrugia, 1997 ▶) and *PLATON* (Spek, 2009 ▶); software used to prepare material for publication: *WinGX* (Farrugia, 1999 ▶) and *PLATON*.

## Supplementary Material

Crystal structure: contains datablock(s) global, I. DOI: 10.1107/S1600536812027006/su2429sup1.cif

Structure factors: contains datablock(s) I. DOI: 10.1107/S1600536812027006/su2429Isup2.hkl

Supplementary material file. DOI: 10.1107/S1600536812027006/su2429Isup3.cml

Additional supplementary materials:  crystallographic information; 3D view; checkCIF report

## Figures and Tables

**Table 1:** Hydrogen-bond geometry (Å, °)

*D*—H⋯*A*	*D*—H	H⋯*A*	*D*⋯*A*	*D*—H⋯*A*
N1—H1⋯O1^i^	0.86	2.02	2.863 (3)	165
N2—H2*A*⋯N2^ii^	0.84 (4)	2.37 (4)	3.192 (4)	167 (3)
N2—H2*B*⋯O1^iii^	0.73 (3)	2.59 (4)	3.230 (3)	147 (4)

Symmetry codes: (i) 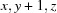; (ii) 
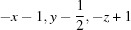
; (iii) 
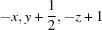
.

**Fig. 1.**
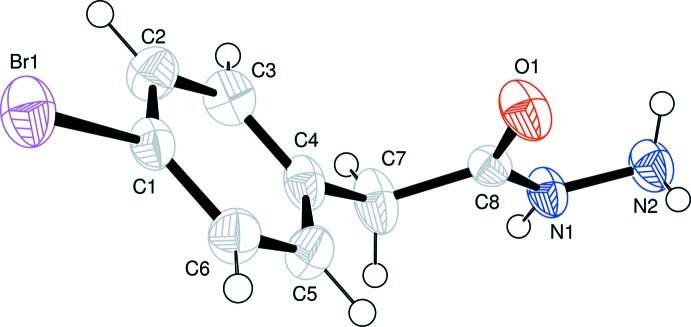
View of the title molecule with the atom numbering. The displacement ellipsoids are drawn at the 50% probability level.

**Fig. 2.**
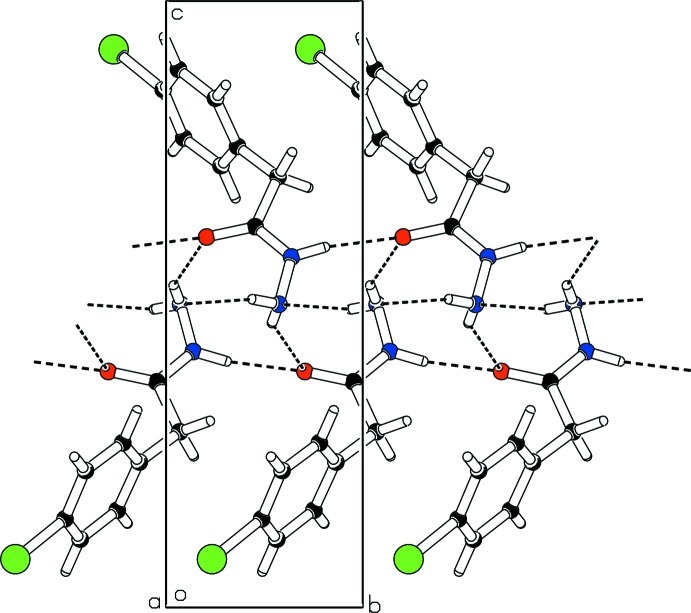
A view along the a axis of the crystal packing of the title compound. The two-dimensional hydrogen bonded network extends in the plane (001). Hydrogen bonds are shown as dashed lines - see Table 1 for details.

## supplementary crystallographic information

### Comment

Recently, we have reported the crystal structure of 2-chlorobenzohydrazide (Ahmad *et al.*, 2012). In continuation of this work we have synthesized the title compound, a hydrazide derivative, and report herein on its crystal structure.

In the title molecule, Fig. 1, the 1-bromo-4-methylbenzene group A (C1–C7/Br1) and the formic hydrazide moiety B (O1/C8/N1/N2) are planar with r. m. s. deviations of 0.0129 Å and 0.0038 Å, respectively. The dihedral angle between these mean planes, A/B, is 80.66 (11)°.

In the crystal, molecules are linked via N—H···O hydrogen bonds to form one-dimensional polymeric chains along [010]. These chains are linked via N-H···N and N-H..O hydrogen bonds to form a two-dimensional polymeric network in (001). The hydrogen bonds give rise to *R*_2_^2^(7) and *R*_3_^2^(7) ring motifs (Bernstein *et al.*, 1995; Table 1 and Fig. 2).

### Experimental

2-(4-Bromophenyl)acetic acid (4.42 g, 0.022 mol) was converted to methyl 2-(4-bromophenyl)acetate by refluxing in methanol (25 ml) in the presence of catalytic amount of sulfuric acid. This ester was then converted into the title compound by refluxing with hydrazine hydrate (80%, 10 ml) in dry methanol. The title compound was purified by recrystallization from dry methanol, giving colourless rod-like crystals [M.p. 438–439 K].

### Refinement

The coordinates of the H-atoms of the NH_2_ group were refined with *U*_iso_(H) = 1.2*U*_eq_(N). The remainder of the H-atoms were included in calculated positions and treated as riding atoms: N–H = 0.86 Å, C–H = 0.93–0.97 Å, with *U*_iso_(H) = 1.2*U*_eq_(N,C).

### Crystal data

**Table:**

C_8_H_9_BrN_2_O	*F*(000) = 228
*M**_r_* = 229.07	*D*_x_ = 1.722 Mg m^−^^3^
Monoclinic, *P*2_1_	Mo *K*α radiation, λ = 0.71073 Å
Hall symbol: P 2yb	Cell parameters from 1677 reflections
*a* = 6.0798 (2) Å	θ = 2.7–28.3°
*b* = 4.8565 (1) Å	µ = 4.60 mm^−^^1^
*c* = 15.1126 (5) Å	*T* = 296 K
β = 98.003 (2)°	Rod, colourless
*V* = 441.88 (2) Å^3^	0.36 × 0.23 × 0.22 mm
*Z* = 2	

### Data collection

**Table:**

Bruker Kappa APEXII CCD diffractometer	1814 independent reflections
Radiation source: fine-focus sealed tube	1677 reflections with *I* > 2σ(*I*)
Graphite monochromator	*R*_int_ = 0.023
Detector resolution: 7.50 pixels mm^-1^	θ_max_ = 28.3°, θ_min_ = 2.7°
ω scans	*h* = −8→8
Absorption correction: multi-scan (*SADABS*; Bruker, 2009)	*k* = −6→4
*T*_min_ = 0.298, *T*_max_ = 0.366	*l* = −20→20
4331 measured reflections	

### Refinement

**Table:**

Refinement on *F*^2^	Secondary atom site location: difference Fourier map
Least-squares matrix: full	Hydrogen site location: inferred from neighbouring sites
*R*[*F*^2^ > 2σ(*F*^2^)] = 0.023	H atoms treated by a mixture of independent and constrained refinement
*wR*(*F*^2^) = 0.059	*w* = 1/[σ^2^(*F*_o_^2^) + (0.024*P*)^2^] where *P* = (*F*_o_^2^ + 2*F*_c_^2^)/3
*S* = 1.06	(Δ/σ)_max_ < 0.001
1814 reflections	Δρ_max_ = 0.44 e Å^−^^3^
115 parameters	Δρ_min_ = −0.47 e Å^−^^3^
1 restraint	Absolute structure: Flack (1983), 583 Friedel pairs
Primary atom site location: structure-invariant direct methods	Flack parameter: 0.007 (11)

### Special details

**Table:**

Geometry. Bond distances, angles *etc*. have been calculated using the rounded fractional coordinates. All su's are estimated from the variances of the (full) variance-covariance matrix. The cell e.s.d.'s are taken into account in the estimation of distances, angles and torsion angles
Refinement. Refinement of *F*^2^ against ALL reflections. The weighted *R*-factor *wR* and goodness of fit *S* are based on *F*^2^, conventional *R*-factors *R* are based on *F*, with *F* set to zero for negative *F*^2^. The threshold expression of *F*^2^ > σ(*F*^2^) is used only for calculating *R*-factors(gt) *etc*. and is not relevant to the choice of reflections for refinement. *R*-factors based on *F*^2^ are statistically about twice as large as those based on *F*, and *R*- factors based on ALL data will be even larger.

### Fractional atomic coordinates and isotropic or equivalent isotropic displacement parameters (Å^2^)

**Table:**

	*x*	*y*	*z*	*U*_iso_*/*U*_eq_	
Br1	0.42290 (4)	0.23371 (7)	0.07962 (1)	0.0435 (1)	
O1	−0.1520 (3)	0.7065 (5)	0.38909 (13)	0.0457 (6)	
N1	−0.2370 (4)	1.1385 (4)	0.42196 (15)	0.0364 (7)	
N2	−0.3296 (5)	1.0741 (6)	0.49999 (17)	0.0420 (8)	
C1	0.2739 (4)	0.4860 (5)	0.14603 (16)	0.0315 (8)	
C2	0.0587 (5)	0.5632 (6)	0.11262 (19)	0.0395 (9)	
C3	−0.0499 (4)	0.7519 (8)	0.16041 (16)	0.0407 (8)	
C4	0.0518 (5)	0.8638 (5)	0.23979 (17)	0.0362 (8)	
C5	0.2669 (5)	0.7798 (7)	0.27159 (17)	0.0416 (12)	
C6	0.3788 (5)	0.5900 (6)	0.22578 (17)	0.0381 (8)	
C7	−0.0693 (6)	1.0694 (6)	0.2896 (2)	0.0494 (10)	
C8	−0.1528 (4)	0.9529 (5)	0.37211 (16)	0.0289 (7)	
H1	−0.23523	1.30826	0.40587	0.0437*	
H2	−0.01156	0.49007	0.05918	0.0474*	
H2A	−0.416 (5)	0.940 (8)	0.491 (2)	0.0503*	
H2B	−0.230 (6)	1.040 (8)	0.531 (2)	0.0503*	
H3	−0.19442	0.80410	0.13848	0.0488*	
H5	0.33752	0.85271	0.32499	0.0500*	
H6	0.52191	0.53407	0.24842	0.0457*	
H7A	−0.19487	1.14063	0.24952	0.0591*	
H7B	0.02941	1.22250	0.30749	0.0591*	

### Atomic displacement parameters (Å^2^)

**Table:**

	*U*^11^	*U*^22^	*U*^33^	*U*^12^	*U*^13^	*U*^23^
Br1	0.0503 (2)	0.0403 (2)	0.0433 (1)	0.0101 (2)	0.0184 (1)	−0.0001 (2)
O1	0.0663 (11)	0.0214 (11)	0.0547 (10)	0.0035 (12)	0.0269 (9)	0.0049 (11)
N1	0.0533 (14)	0.0226 (11)	0.0367 (11)	0.0007 (9)	0.0184 (10)	0.0019 (8)
N2	0.0511 (16)	0.0378 (14)	0.0407 (14)	0.0000 (12)	0.0193 (12)	−0.0020 (11)
C1	0.0367 (13)	0.0284 (14)	0.0315 (12)	0.0012 (10)	0.0123 (10)	0.0007 (10)
C2	0.0393 (14)	0.0426 (17)	0.0356 (13)	0.0034 (12)	0.0021 (12)	−0.0016 (12)
C3	0.0373 (11)	0.0407 (16)	0.0442 (12)	0.0084 (16)	0.0063 (10)	0.0062 (17)
C4	0.0523 (16)	0.0247 (13)	0.0352 (13)	0.0029 (11)	0.0185 (12)	0.0034 (10)
C5	0.0488 (14)	0.044 (3)	0.0321 (11)	−0.0038 (14)	0.0061 (11)	−0.0051 (12)
C6	0.0344 (14)	0.0440 (16)	0.0355 (13)	0.0028 (12)	0.0040 (12)	0.0009 (12)
C7	0.077 (2)	0.0298 (16)	0.0479 (17)	0.0083 (15)	0.0319 (16)	0.0057 (13)
C8	0.0312 (12)	0.0221 (13)	0.0338 (12)	0.0001 (10)	0.0064 (10)	−0.0008 (9)

### Geometric parameters (Å, º)

**Table:**

Br1—C1	1.893 (2)	C4—C5	1.390 (4)
O1—C8	1.224 (3)	C4—C7	1.503 (4)
N1—N2	1.411 (4)	C5—C6	1.387 (4)
N1—C8	1.323 (3)	C7—C8	1.520 (4)
N1—H1	0.8600	C2—H2	0.9300
N2—H2B	0.73 (3)	C3—H3	0.9300
N2—H2A	0.84 (4)	C5—H5	0.9300
C1—C2	1.387 (4)	C6—H6	0.9300
C1—C6	1.379 (4)	C7—H7A	0.9700
C2—C3	1.389 (4)	C7—H7B	0.9700
C3—C4	1.382 (4)		
			
N2—N1—C8	123.8 (2)	O1—C8—C7	123.0 (2)
C8—N1—H1	118.00	N1—C8—C7	114.4 (2)
N2—N1—H1	118.00	O1—C8—N1	122.5 (2)
N1—N2—H2B	101 (3)	C1—C2—H2	121.00
H2A—N2—H2B	112 (4)	C3—C2—H2	121.00
N1—N2—H2A	111 (2)	C2—C3—H3	119.00
Br1—C1—C2	118.61 (19)	C4—C3—H3	119.00
Br1—C1—C6	120.2 (2)	C4—C5—H5	119.00
C2—C1—C6	121.2 (2)	C6—C5—H5	119.00
C1—C2—C3	118.8 (2)	C1—C6—H6	121.00
C2—C3—C4	121.5 (3)	C5—C6—H6	121.00
C3—C4—C7	120.3 (3)	C4—C7—H7A	109.00
C3—C4—C5	118.1 (3)	C4—C7—H7B	109.00
C5—C4—C7	121.6 (3)	C8—C7—H7A	109.00
C4—C5—C6	121.7 (3)	C8—C7—H7B	109.00
C1—C6—C5	118.7 (3)	H7A—C7—H7B	108.00
C4—C7—C8	114.0 (2)		
			
N2—N1—C8—O1	1.3 (4)	C2—C3—C4—C7	179.5 (3)
N2—N1—C8—C7	178.1 (3)	C3—C4—C5—C6	0.1 (4)
Br1—C1—C2—C3	−178.9 (2)	C7—C4—C5—C6	179.9 (3)
C6—C1—C2—C3	1.0 (4)	C3—C4—C7—C8	104.7 (3)
Br1—C1—C6—C5	178.4 (2)	C5—C4—C7—C8	−75.1 (4)
C2—C1—C6—C5	−1.5 (4)	C4—C5—C6—C1	1.0 (4)
C1—C2—C3—C4	0.2 (5)	C4—C7—C8—O1	−10.6 (4)
C2—C3—C4—C5	−0.7 (4)	C4—C7—C8—N1	172.7 (2)

### Hydrogen-bond geometry (Å, º)

**Table:**

*D*—H···*A*	*D*—H	H···*A*	*D*···*A*	*D*—H···*A*
N1—H1···O1^i^	0.86	2.02	2.863 (3)	165
N2—H2*A*···N2^ii^	0.84 (4)	2.37 (4)	3.192 (4)	167 (3)
N2—H2*B*···O1^iii^	0.73 (3)	2.59 (4)	3.230 (3)	147 (4)

Symmetry codes: (i) *x*, *y*+1, *z*; (ii) −*x*−1, *y*−1/2, −*z*+1; (iii) −*x*, *y*+1/2, −*z*+1.
